# Impact of timing of adjuvant chemotherapy initiation on survival for elderly patients with stage III colon cancer in Norway – a register-based cohort study

**DOI:** 10.1186/s12885-025-14942-y

**Published:** 2025-10-14

**Authors:** Elin Marthinussen Gustavsen, Stig Norderval, Liv Marit Dørum, Eva Stensland, Ellinor Haukland, Yohannes Tesfay, Beate Hauglann

**Affiliations:** 1 Department of Community Medicine, The Arctic University of Norway (UiT), Tromsø, Norway; 2 Centre for Clinical Documentation and Evaluation (SKDE), Northern Norway Regional Health Authority, Tromsø, Norway; 3 Department of Gastrointestinal Surgery, University Hospital of Northern Norway, Tromsø, Norway; 4 Department of Clinical Medicine, Faculty of Health Sciences, The Arctic University of Norway (UiT), Tromsø, Norway; 5 Cancer Registry of Norway, Norwegian Institute of Public Health, Oslo, Norway; 6 Department of Oncology and Palliative Medicine, Nordland Hospital, Bodø, Norway; 7 SHARE – Center for Resilience in Healthcare, Faculty of Health Sciences, Department of Quality and Health Technology, University of Stavanger, Stavanger, Norway

**Keywords:** Colon cancer, Adjuvant chemotherapy, Timing, Survival, Elderly

## Abstract

**Background:**

Standard treatment for stage III colon cancer is major surgical resection followed by adjuvant chemotherapy (ACT). Norwegian guidelines recommend initiation of ACT within 4–6 weeks after resection, but consensus regarding optimal timing of ACT is lacking.

**Objective:**

To investigate the impact of ACT timing on 5-year overall survival (OS) of elderly patients with stage III colon cancer in Norway.

**Methods:**

This population-based retrospective cohort study included patients aged 70 years or older who underwent major surgical resection for stage III colon cancer diagnosed between 2011 and 2021. Patients were grouped by ACT timing after resection: ≤ 6, 7–8, 9–10, and 11–13 weeks, in addition to those with resection only. The 5-year OS was assessed using Kaplan-Meier analysis and Cox proportional hazards models.

**Results:**

Among 4 075 patients included, 1 408 were provided ACT. Median timing of ACT was 6.4 weeks after resection. Initiation of ACT in weeks 7–10 after resection was not associated with increased mortality risk compared to initiation within the first 6 weeks. Delaying the initiation of ACT beyond 10 weeks after resection was associated with worse 5-year OS (hazard ratio 1.49, 95% confidence interval 1.04–2.12). The association between survival benefit and ACT timing varied based on risk levels: For low risk patients, there was an association of improved survival benefit when ACT was initiated within 8 weeks after resection compared to resection alone, whereas for high risk patients, the association of survival benefit was better for those provided ACT in weeks 9–10 as well.

**Conclusions:**

Our findings support initiation of ACT within 8 weeks after major resection to maximise survival benefits in elderly patients with stage III colon cancer. However, for certain patient groups, initiation of ACT seems beneficial even up to 10 weeks after resection. The findings suggest greater flexibility in ACT initiation timing, benefiting both patients and health care services.

## Introduction

Colon cancer is one of the most commonly diagnosed cancers globally. The highest rates occur in developed regions such as Europe, Australia, and Northern America [1]. In Norway, colon cancer is the fourth most common cancer type [2]. Of the 3 536 persons who were diagnosed with colon cancer in Norway in 2023, 61% were aged 70 years or older [3, 4]. Given that colon cancer risk increases with age [5], this proportion is expected to increase to 72% by 2040 due to the aging population [6].

Standard treatment for patients with stage III colon cancer involves major surgical resection followed by adjuvant chemotherapy (ACT), a well-established regimen since the National Institutes of Health consensus in 1990 [7]. The consensus was based on clinical trials that demonstrated survival benefits with ACT [8, 9]. Randomized studies conducted in Norway, Sweden, and Denmark during the 1990 s supported these findings [10, 11].

The optimal time frame for the initiation of ACT has not yet been established by clinical trials. Nonetheless, several studies have found increased mortality among patients where ACT was initiated more than 8 weeks after resection [12–14]. A meta-analysis of eight studies comparing delayed ACT to standard care, confirmed that initiation of ACT beyond 8 weeks after resection was associated with worse overall survival (OS) [15]. Norwegian guidelines recommend initiation of ACT for stage III colon cancer patients younger than 75 years of age within 4–6 weeks after resection [16].

Elderly patients are often underrepresented in clinical trials [17]. However, a pooled analysis of seven clinical trials, along with several cohort studies, have shown survival benefit from ACT also in elderly patients [18–23]. According to Norwegian guidelines, functional status, comorbidities, and overall health should be assessed before initiation of ACT to patients aged 75 years or older. The recommended timing of ACT for these patients, if deemed fit for the treatment, is the same as for younger patients.

There is a shortage of information regarding the impact of ACT timing on elderly colon cancer patients. A study has shown that the initiation of ACT in Norwegian patients aged ≥ 70 years was as common in weeks 7 and 8 as within the first 6 weeks after resection [24]. The aim of the present study was to investigate whether delayed initiation of ACT beyond the 6 weeks recommended in the current Norwegian guidelines, affects OS in elderly patients with stage III colon cancer.

## Methods

### Data sources

This register-based cohort study utilised individual-level data from mandatory health and administrative national registries with complete coverage. The data linkage was enabled by encrypted serial numbers generated from the personal identity number assigned to all Norwegian citizens.

The Cancer Registry of Norway (CRN) is a comprehensive national registry with mandatory reporting of all cancer cases, ensuring high data quality and completeness [25]. For this study, the CRN provided data on all patients aged 70 years or older diagnosed with stage III colon cancer in the study period. The dataset included information on age, sex, type of cancer, date of diagnosis, and basis of diagnosis. Additionally, data on pathological stage and major resection were obtained from the Norwegian Colorectal Cancer Registry. This is a specialised clinical sub-registry of the CRN, which has collected data systematically on colon cancer since 2007.

Data on the use of specialised health care services, including diagnoses, procedures, treating hospitals, and visit details, were retrieved from the Norwegian Patient Registry. Data on the use of primary health care services, including contact dates and International Classification of Primary Care (ICPC) codes, were obtained from the Norwegian Control and Payment of Health Reimbursements Database.

Furthermore, Statistics Norway, the national statistical office, provided individual-level annual demographic and socioeconomic data, including details on residence, education, income, and household type.

### Study population

**Fig. 1:**
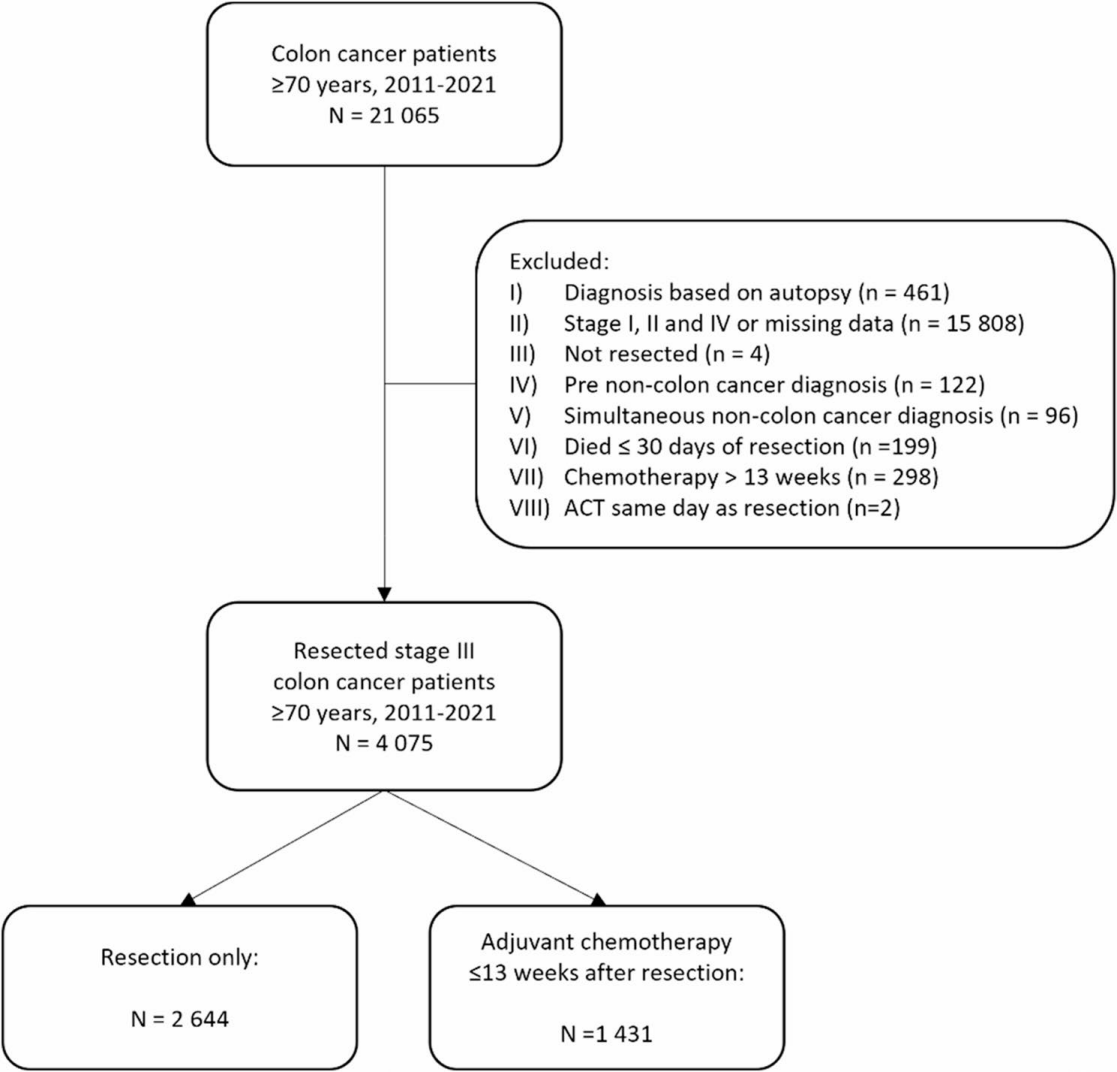
Flowchart showing the inclusion of patients in the study

This study included all Norwegian residents aged 70 years or older diagnosed with stage III colon cancer from 1 January 2011 to 31 December 2021, and who had undergone major resection of the cancer (Fig. 1).

Colon cancer patients were identified in the CRN data using the International Classification of Diseases 10th Revision (ICD-10) code C18. Stage III colon cancer was defined in the Norwegian Colorectal Cancer Registry data according to the pathological tumour-node-metastasis (TNM) classification: any pT, pN1-2 and pM0. This includes all cases where the tumour has spread to regional lymph nodes (N1-2), but not to distant metastatic sites (M0). ACT was identified in by the diagnosis code Z51.1* or the procedural codes WBOC05, WBOC08 or WBOC20 in data from the Norwegian Patient Registry.

The exclusion criteria comprised: (1) patients diagnosed with non-colon cancer prior to or concurrent with the colon cancer diagnosis, (2) patients who died within 30 days after resection, (3) patients who received ACT same day as resection, and (4) patients who initiated chemotherapy more than 13 weeks after resection were excluded from analysis, as chemotherapy initiated beyond this period is likely administrated for reasons other than adjuvant therapy.

### Definitions

Time to initiation of ACT was calculated as the number of days from the date of major surgical resection to the date of the first session of chemotherapy and grouped into five categories: 1–6, 7–8, 9–10, and 11–13 weeks after resection and no ACT after surgical resection. The clinical characteristics included cancer risk level (low: T1–T3N1; and high: T1–T3N2, T4N+), and urgency of resection (elective or acute).

Frailty was measured by a frailty index (FI) based on primary care data [26]. ICPC codes from the 12 months preceding the cancer diagnosis were used to calculate the individual FI. The FI was thereafter categorised into three scores: low (0–1), intermediate (2–3), and high score (≥ 4).

Patient demographics included sex, age at diagnosis (categorised into three groups: 70–74, 75–79, and ≥ 80 years), household type (categorised as living alone, living with a cohabitant, or residing in non-private household such as nursing facilities) and socioeconomic status (SES). The patient’s SES was measured as a composite score based on a weighted combination of education and personal income [27]. The patient’s highest education level was categorised into to four levels: primary education (< 10 years), upper secondary/vocational, undergraduate degree and postgraduate degree. Yearly after-tax personal income for the year before diagnosis was adjusted for the consumer price index and divided into sex-specific quartiles. Empirically derived weights for individuals aged ≥ 66 years from Lindberg et al. [27] were applied to combine education and income levels into a composite score categorised as low, intermediate, and high SES.

### Statistics

Descriptive statistics were used to describe characteristics across different timings of ACT. The variables were compared using chi-squared tests.

Survival time was defined as the period from the date of resection to death. Patients were censored at the end of the 5-year follow-up period, upon emigration, diagnosis of non-colon cancer following the initial colon cancer diagnosis, or at the follow-up cut-off date (1st January 2023).

Kaplan–Meier curves from univariate survival analysis were used to compare 5-year OS among patients receiving ACT, segmented by the timing of ACT initiation, and those with resection only. The differences between the survival curves were assessed using log-rank tests.

The relationship between the timing of ACT and 5-year OS was analysed using multivariable Cox proportional hazards regression models, adjusting for multiple survival-related factors. Age-stratified analysis was conducted for patients aged 70–74 years and ≥ 75 years. P-values below 0.05 were considered statistically significant. All models were examined for multicollinearity by inspecting correlation coefficients and variance inflation factors.

The primary analysis included resected patients who had received ACT. In this analysis, patients who initiated chemotherapy within 6 weeks after resection served as the reference group. Comparisons were made between this reference group and patients who initiated ACT at later time points. In a secondary analysis, we expanded the study by including resected patients who did not receive ACT. This analysis compared patients who received ACT at different time points to those who did not receive ACT (reference group).

To address potential immortal time bias [28], landmark analyses using both Kaplan-Meier and multivariable Cox proportional hazards regression were conducted. These analyses compared patients who received ACT within a specific landmark (1–6 weeks, 7–8 weeks, 9–10 weeks, and 11–13 weeks after resection) to those who had not received ACT by that landmark. Patients who received ACT after that landmark were censored from the analysis from the period they received ACT.

Data were analysed using SAS V.9.4 (SAS Institute).

## Results

During the period 2011–2021, 4 075 patients aged 70 years or older, diagnosed with stage III colon cancer and who underwent major resection, were eligible for inclusion in this study (Table 1). Of them, 1 431 received ACT and median age of these patients was 74 years (range 70 to 88 years). The median age of patients who did not received ACT was 82 years (range 70 to 99 years).

**Table 1.**
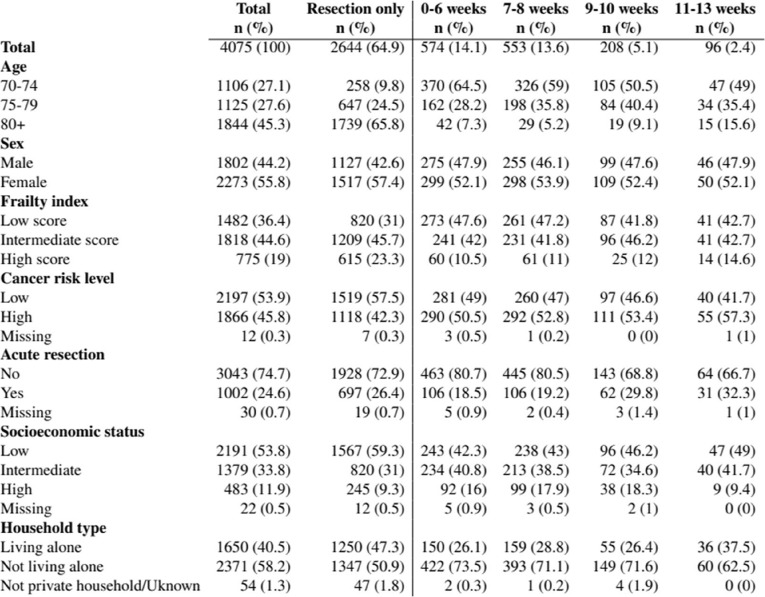
Characteristics of elderly patients (≥70 years) diagnosed with stage III colon cancer in Norway in 2011–2021 and who had undergone major resection, in total and by timings of adjuvant chemotherapy

**Fig. 2:**
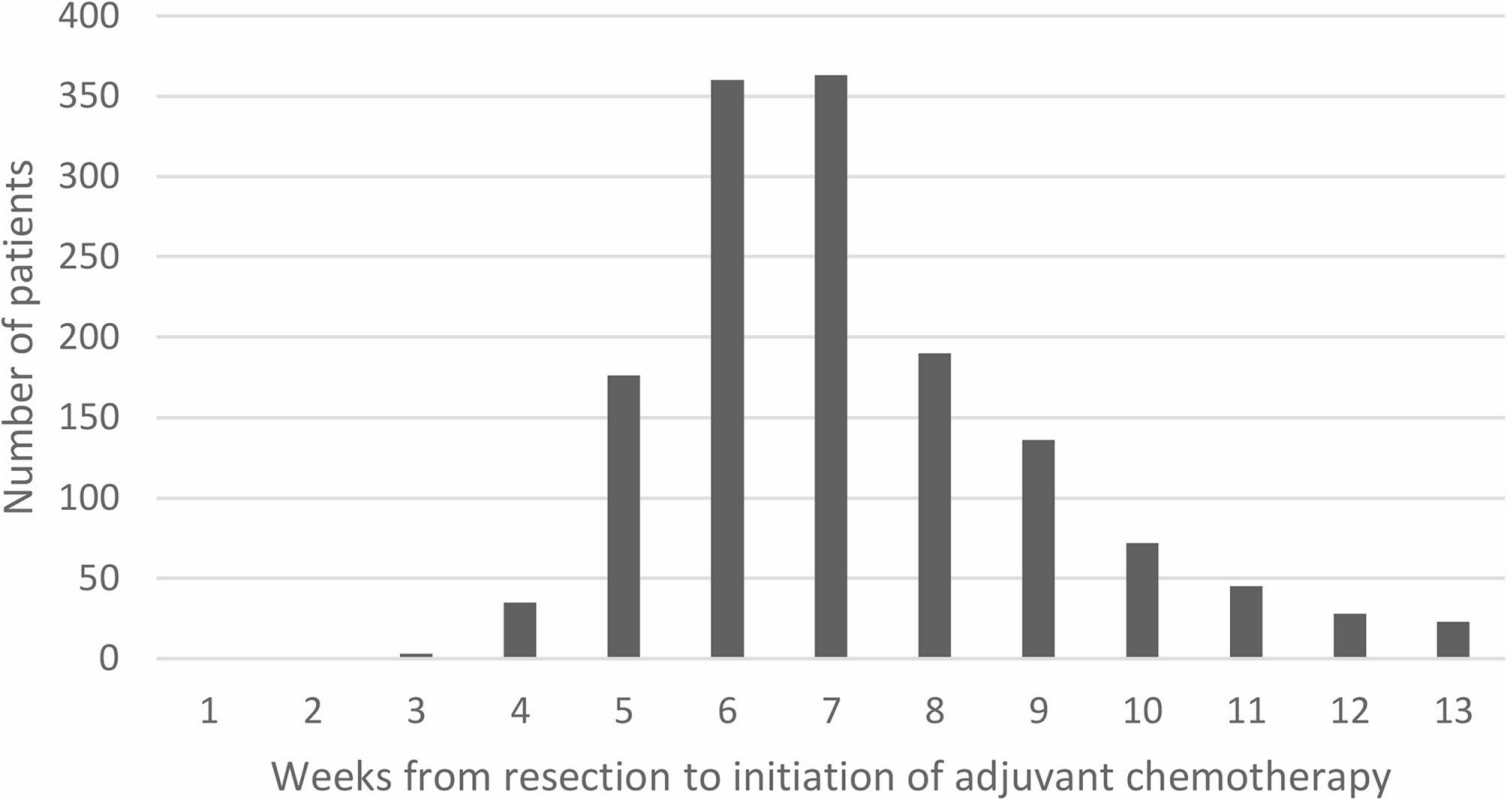
Distribution of the time (weeks) from resection to adjuvant chemotherapy for elderly patients (≥70 years) diagnosed with stage III colon cancer in Norway in 2011–2021

Median timing of ACT was 6.4 weeks after resection. Treatment was initiated within 6 weeks in 40% (*n* = 574), during weeks 7–8 in 39% (*n* = 553), during weeks 9–10 in 15% (*n* = 208), and during weeks 11–13 in 7% (*n* = 96). The timing of ACT in weeks from resection is presented in Fig. 2. Patients who received their first ACT during weeks 11–13 differed somewhat from those who received ACT earlier, with a significantly higher proportion of patients who were 80 years or older, were living alone, and had acute resection.

**Fig. 3:**
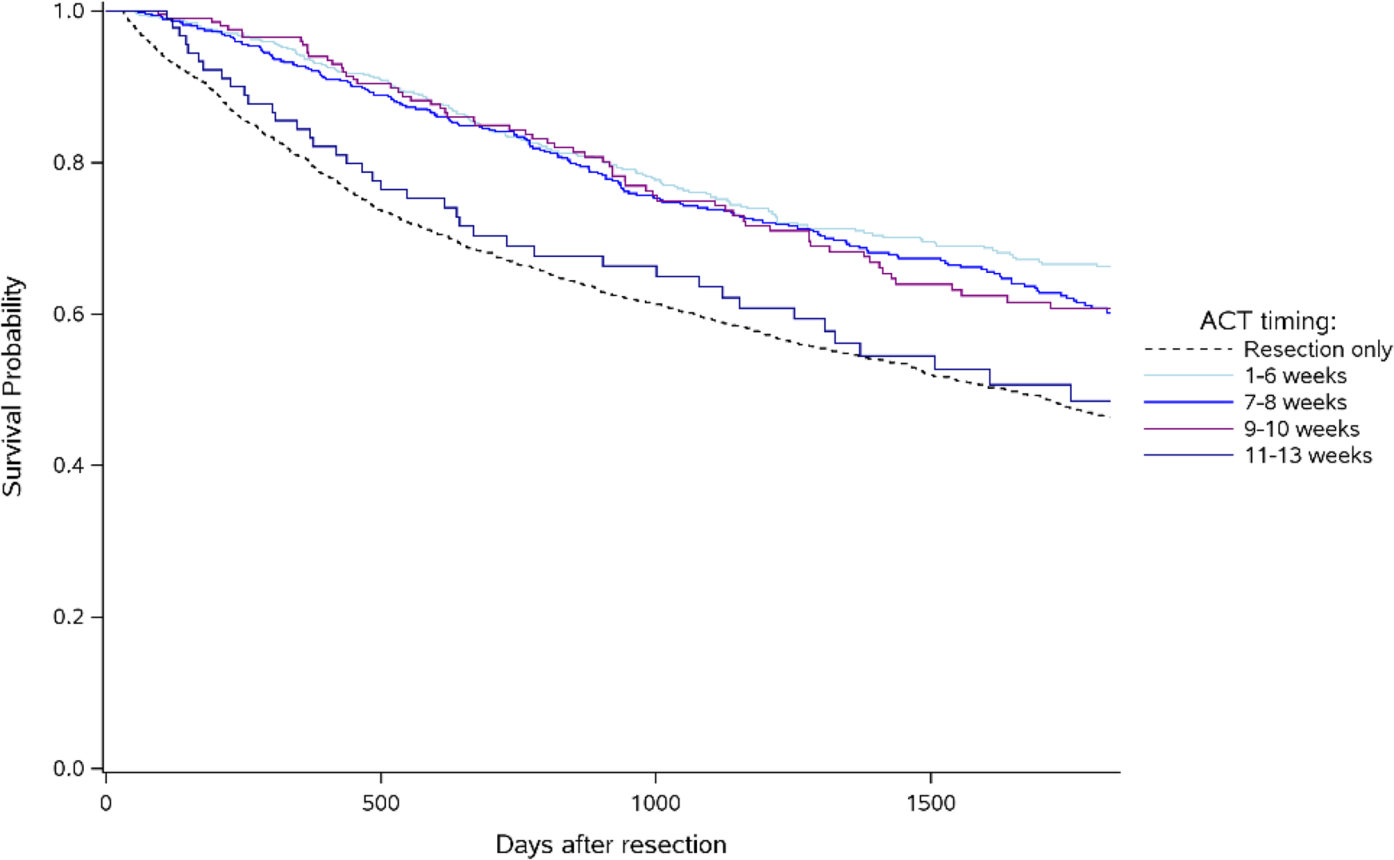
Crude 5-year overall survival stratified by timing of adjuvant chemotherapy in addition to the survival curve of patients with resection only

Kaplan-Meier 5-year survival curves, stratified by ACT initiation are shown in Fig. 3, including the resection only category. The observed crude 5-year OS rate was 72%, 68%, 69%, and 58% for patients who received their first ACT within ≤ 6 weeks, during weeks 7–8, weeks 9–10 and weeks 11–13, respectively, and 55% for those who had resection only.

### Timing of ACT

The unadjusted Cox proportional hazard regression showed that initiation of ACT later than 10 weeks after resection was significantly associated with an increased hazard of death compared to initiation within 6 weeks (hazard ratio (HR) 1.83, 95% confidence interval (CI) 1.29–2.59) (Table 2). ACT initiated during weeks 7–8 and 9–10 after resection was not associated with increased mortality risk compared to initiation within 6 weeks.

**Table 2.**
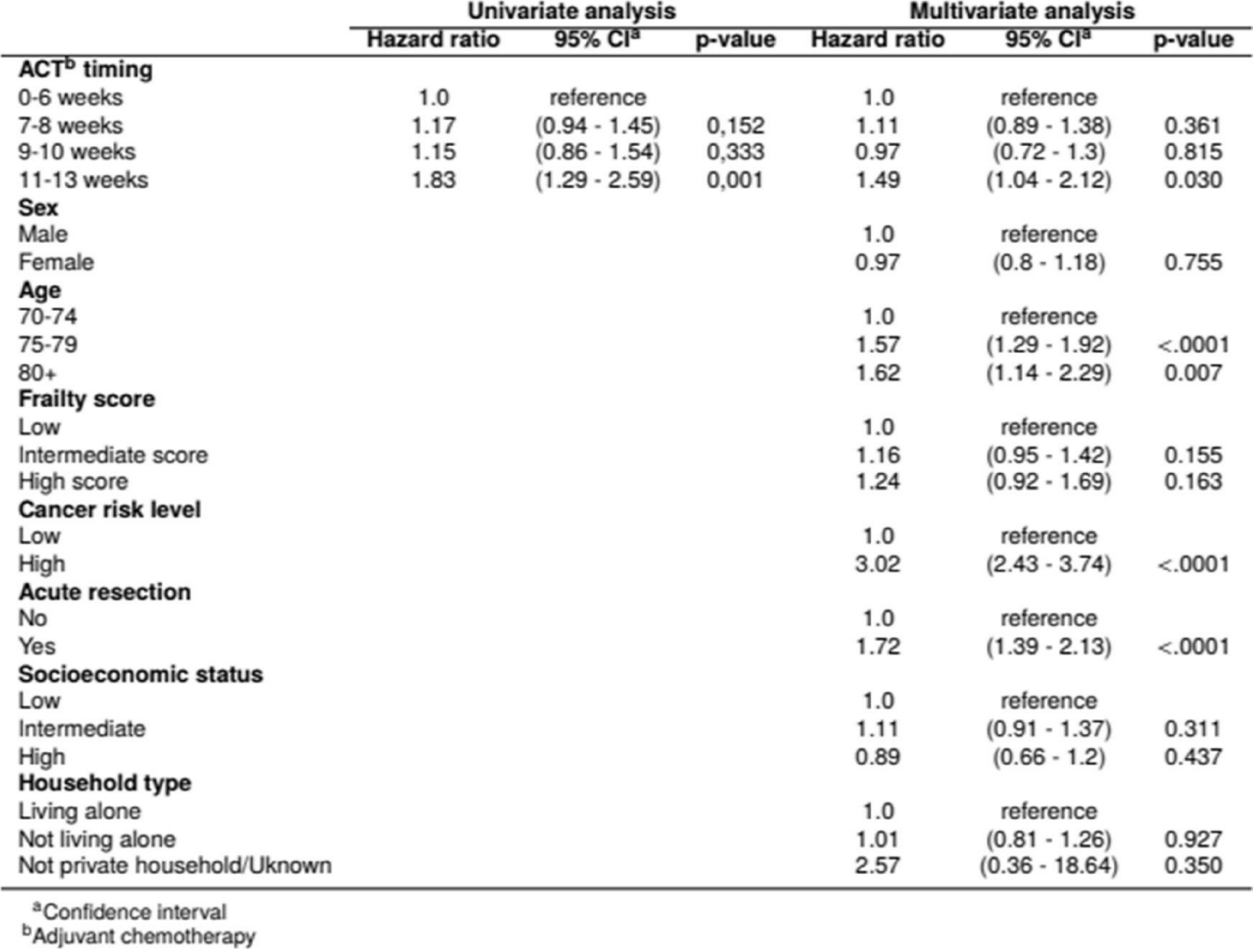
Univariate and multivariate hazard ratios of death among elderly patients (≥70 years) with both resection and had been provided adjuvant chemotherapy for stage III colon cancer in Norway

In the fully adjusted model, the results were similar to the univariate analysis (Table 2): Initiation of ACT later than 10 weeks after resection was significantly associated with an increased hazard of death compared to initiation within 6 weeks (HR 1.49, 95% CI: 1.04–2.12), whereas initiation during weeks 7–8 and 9–10 after resection was not (7–8 weeks: HR 1.11, 95% CI: 0.89–1.38; 9–10 weeks: HR 0.97, 95% CI: 0.72–1.30).

Stratifying the analysis by two age groups – 70–74 years and ≥ 75 years – did not alter the overall results. The effect estimates for the timing of ACT were generally consistent with the overall analysis.

### ACT compared to resection only

Patients not receiving ACT differed from those who received ACT. The “resection only” patient group had a significantly higher proportion having high frailty, low risk level, acute resection, and being 80 years or older compared to those receiving ACT. For instance, among patients who underwent resection only, 23% had high frailty, compared to 11% of those who received ACT. Similarly, 66% of patients who did not receive ACT were aged 80 years or older, compared to 7% of those who received ACT. The “resection only” group also had a higher proportion of females, low SES, and patients living alone compared to those receiving ACT.

In the fully adjusted Cox regression model, the hazard of death of ACT initiation during weeks 11–13 after resection, was not significantly different from that of patients with resection only (HR 0.97, 95% CI: 0.70–1.35) (Table 3). ACT initiated during weeks 1–6, 7–8, and 9–10 was significantly associated with a decreased hazard of death compared to the resection only category.

**Table 3.**
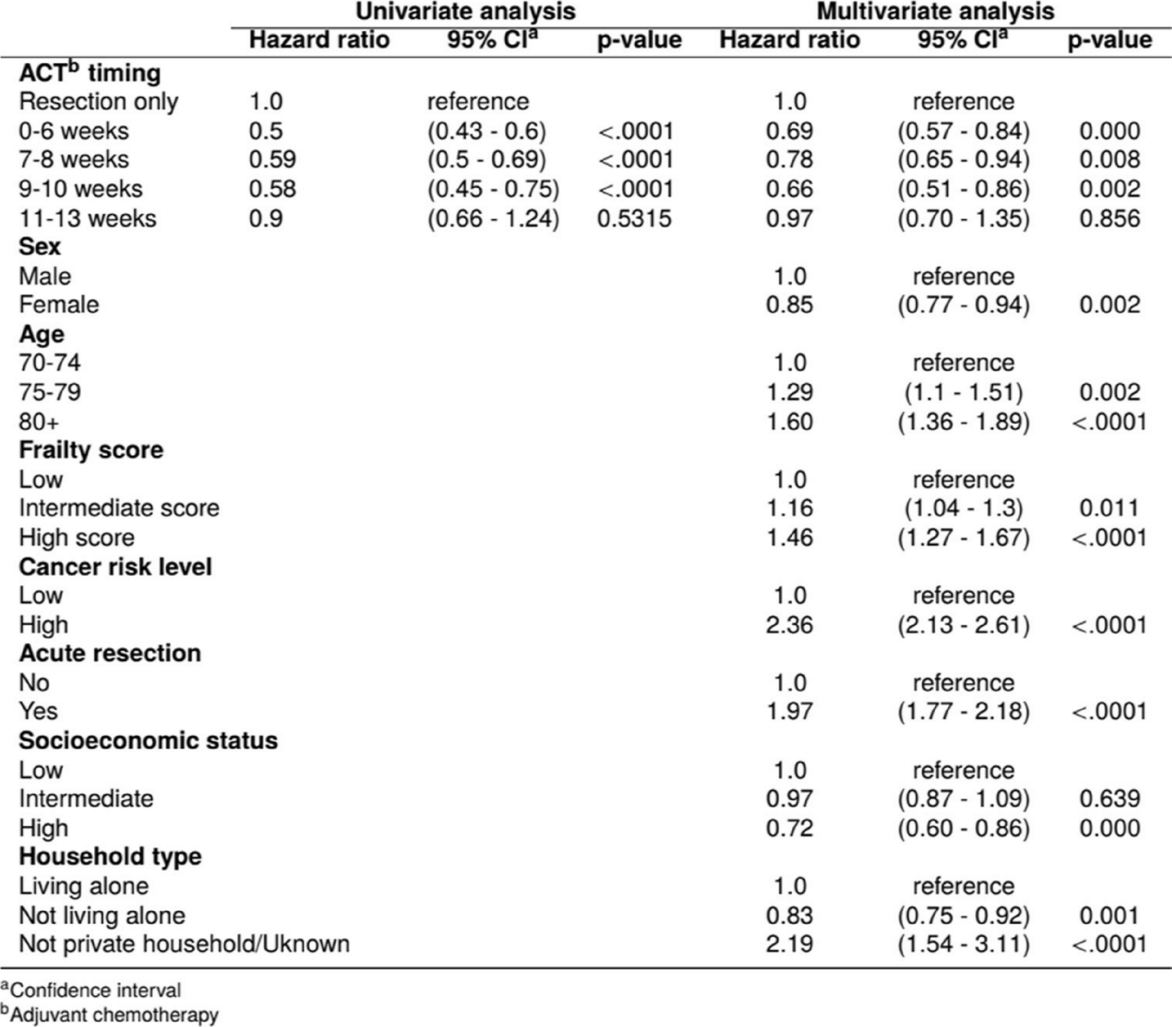
Univariate and multivariate hazard ratios of death among all elderly patients (≥70 years) with stage III colon cancer in Norway

When stratified by two age groups, 70–74 years and ≥ 75 years, the effect estimates for the timing of ACT were generally consistent with the overall analysis.

### Immortal time bias

**Fig. 4:**
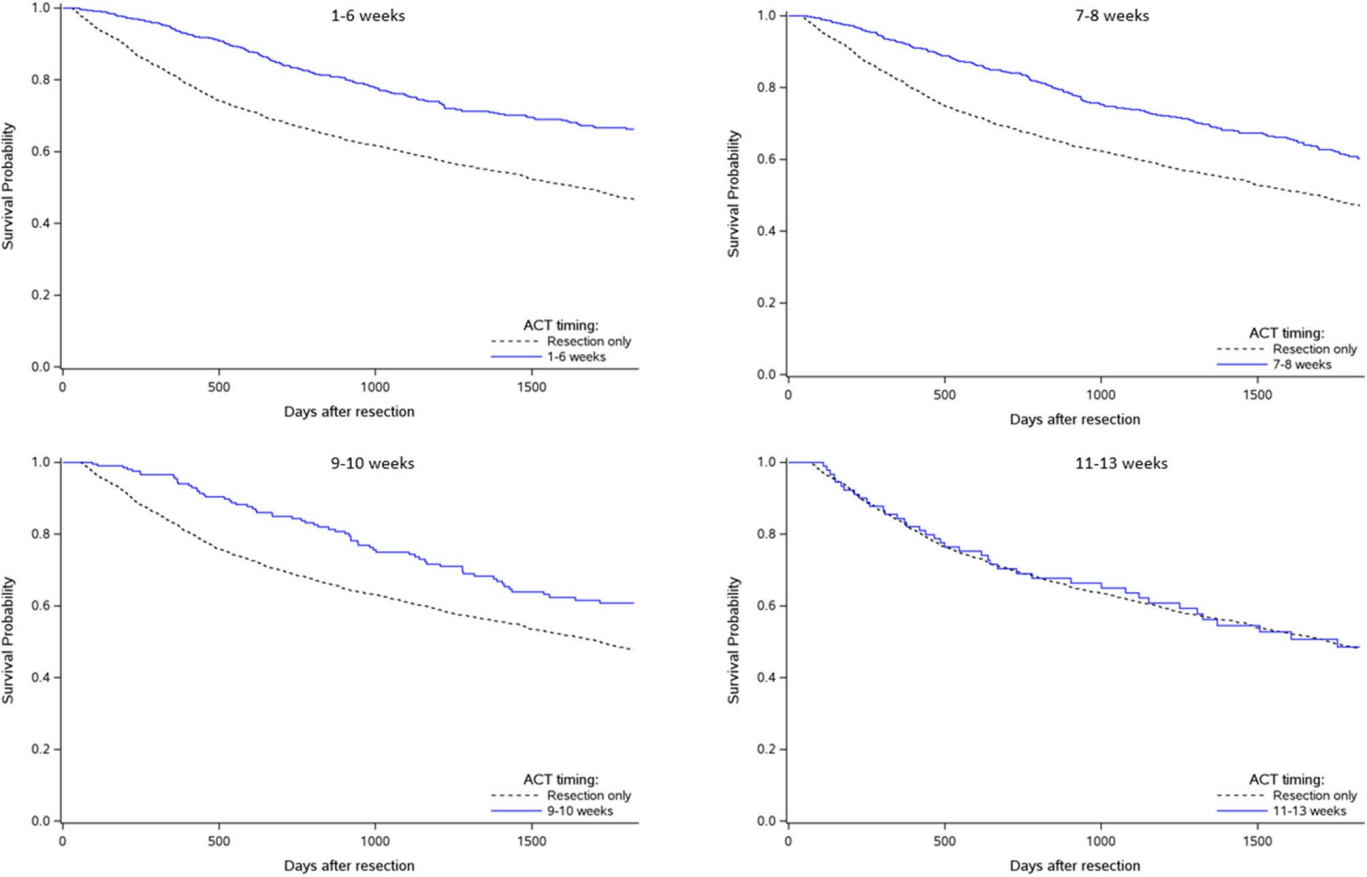
Immortal time bias corrected 5-year overall survival for adjuvant chemotherapy initiated in weeks 1–6 (upper left), 7–8 (upper right), 9–10 (lower left), and 11–13 (lower right) compared to resection only

Landmark analyses were conducted to correct for immortal time bias. These analyses compared patients who received ACT during specific landmarks (1–6 weeks, 7–8 weeks, 9–10 weeks, and 11–13 weeks after resection) to those who had not been provided ACT by those landmarks. Figure 4 shows Kaplan-Meier 5-year survival curves, corrected for immortal time bias, comparing categories of ACT timing to the resection only category. All initiation timings, except for weeks 11–13 after resection, had significantly better survival probability compared to resection only.

**Fig. 5:**
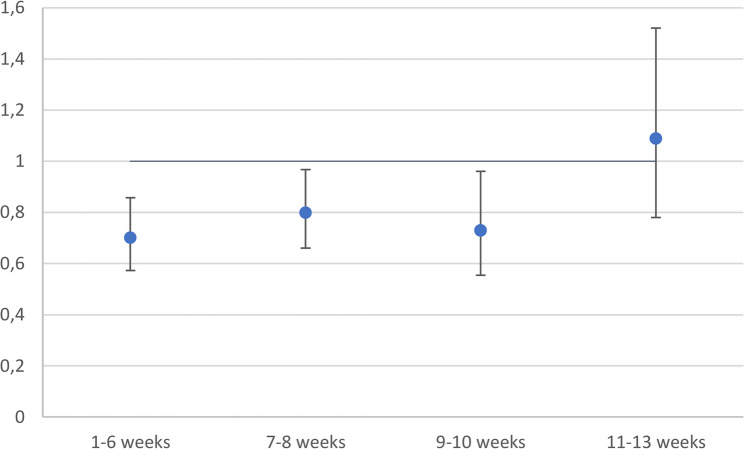
Immortal time bias corrected hazard ratios of death for the timing of adjuvant chemotherapy compared to resection only

In the fully adjusted Cox regression model corrected for immortal time bias, the hazard of death was similar for patients provided ACT during weeks 11–13 and patients having resection only (Fig. 5). ACT initiation during weeks 1–6, 7–8, and 9–10 were all significantly associated with a decreased hazard of death compared to resection only.

### Cancer risk level

**Fig. 6:**
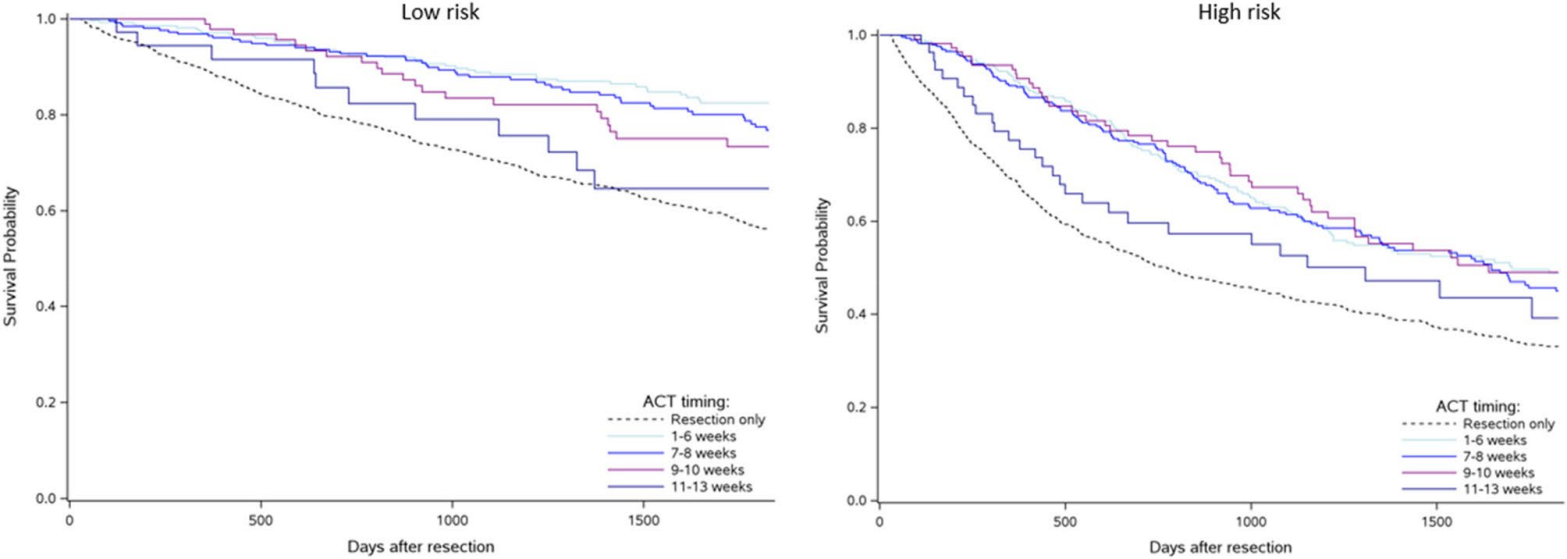
Crude 5-year overall survival stratified by timing of adjuvant chemotherapy in addition to the survival curve of patients with resection only for elderly patients with low risk cancer (left) and high risk cancer (right)

Kaplan-Meier 5-year survival curves by risk level are shown in Fig. 6. The observed crude 5-year OS rates were lower for patients with high risk compared to those with low risk. The observed crude 5-year OS rates for resection only were 48% for high risk and 64% for low risk patients. For those receiving ACT, rates were 59% vs. 86% within ≤ 6 weeks, 56% vs. 82% during weeks 7–8, 60% vs. 78% during weeks 9–10, and 48% vs. 77% during weeks 11–13 for high risk vs. low risk patients, respectively.

**Fig. 7:**
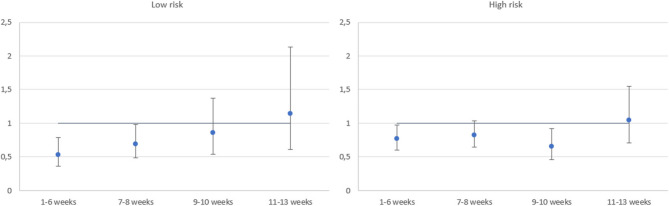
Immortal time bias corrected hazard ratios of death for the timing of adjuvant chemotherapy compared to resection only for elderly patients with low risk cancer (left) and high risk cancer (right)

In the fully adjusted Cox regression model, corrected for immortal time bias, the hazard ratio of death across the ACT timings varied somewhat between the two risk levels (Fig. 7). For low risk patients, initiation of ACT during weeks 1–6 and 7–8 was significantly associated with decreased hazard of death compared to resection only (1–6 weeks: HR 0.53, 95% CI: 0.36–0.78; 7–8 weeks: HR 0.69, 95% CI: 0.49–0.98). For high risk patients, ACT during weeks 1–6 and 9–10 was significantly associated with decreased hazard of death, with weeks 7–8 being borderline significant: (1–6 weeks: HR 0.77, 95% CI: 0.61–0.98; 7–8 weeks: HR 0.82, 95% CI: 0.65–1.03; 9–10 weeks: HR 0.65, 95% CI: 0.46–0.92).

When stratified by two age groups, 70–74 years and ≥ 75 years, the effect estimates for the timing of ACT of the two risk levels were generally consistent with the overall analyses. However, for patients with low risk level, the younger patients, 70–74 years, receiving ACT during weeks 9–10 was also significantly associated with decreased hazard of death compared to resection only.

## Discussion

This retrospective cohort study assessed the timing of ACT and its impact on 5-year OS in elderly patients with stage III colon cancer in Norway. Our findings indicate that ACT initiation during weeks 7–8 and 9–10 after resection is not associated with increased mortality risk compared to initiation within the first 6 weeks. However, delaying the initiation beyond 10 weeks after resection is related to increased mortality risk. The association between survival benefit and ACT timing differ somewhat between the cancer risk levels. Sensitivity analyses stratified by age, indicate that results for patients aged less than 75 years and those aged 75 years or older are generally consistent with the overall analysis, supporting the robustness of our findings.

### Guidelines

Our study shows that the majority (60%) of the patients provided ACT commence ACT later than 6 weeks after resection, in contrast to Norwegian guidelines that recommend initiation within 4 to 6 weeks. Nevertheless, the observed 5-year OS rates are relatively stable for patients with an initiation of ACT within 10 weeks after surgery (68–72%). Furthermore, the fully adjusted model shows that initiation of ACT during 7–10 weeks after resection is not associated with an increased mortality risk compared to initiation within the first 6 weeks. Previous studies have found that initiation of ACT within 8 weeks after resection was not related to worse OS, while delaying it beyond 8 weeks was [12–14, 29, 30]. However, these studies used different cut-offs of the initiation of ACT, e.g. comparing 9–12 weeks to the first 4 weeks or simply comparing > 8 weeks to ≤ 8 weeks.

Our findings suggest a degree of flexibility in the timing of ACT initiation compared to the national recommendations. This would provide a substantial advantage to the health care services by allowing additional time for treatment planning and would be especially beneficial in managing elderly patients who might need more time for recovery before starting chemotherapy [31]. An extension in the ACT timing would enable health care providers to better address each patient’s unique needs, ensuring that every case receives the necessary attention and resources. Such flexibility is especially important given the increasing number of cancer patients and the growing demands on oncology departments.

Our results would also be valuable for cancer patients, as treatment delays may cause psychological distress. A qualitative study found that patients felt an urgent need to initiate their cancer treatment soon after their diagnosis, preferring shorter time frames than those recommended in the guidelines [32]. The patients expressed a fear of both cancer progression while awaiting care and of poor outcomes of the delayed treatment.

### ACT compared to resection only

Patients not receiving ACT differ from those who received ACT. They are for instance typically older, live alone, and have higher frailty index scores than those provided ACT. These characteristics might have influenced the decision against aggressive chemotherapy due to concerns about potential toxicity and reduced tolerance. This underscores the complexity of cancer care in elderly populations, where the benefits of treatment must be carefully weighed against the risks and overall health status of the patient. A multidisciplinary approach is essential to address these complex needs in a comprehensive way [33, 34].

In our study, initiation of ACT within 10 weeks after resection is associated with a greater survival benefit compared to resection only. However, the association is not sustained when ACT is initiated beyond 10 weeks after resection. Other studies have found a survival benefit with even longer timespans between resection and ACT. Turner et al. [35] found that initiation of ACT more than 24 weeks after resection improved the overall survival compared to those with resection only. Similarly, Gao et al. [30] showed a survival benefit in patients ≥ 66 years old when ACT was initiated up to 21 weeks after resection compared to those with resection only.

### Cancer risk level

Stage III colon cancer is typically divided into low and high risk level based on tumour characteristics. This risk stratification guides treatment decisions, especially concerning the duration of ACT [36]. Our study finds that the association between survival benefit and ACT timing is somewhat different between those with low and high risk level: For low risk patients, there is an association of improved survival benefit when ACT is initiated within 8 weeks after resection compared to those with resection only, whereas for high risk patients, the association of survival benefit is better for those provided ACT in weeks 9–10 as well. To our knowledge, this is the first study to explore the impact of ACT timing across the two risk levels.

### ACT initiated 11–13 weeks after resection

A notable decline in survival is observed in patients receiving ACT during weeks 11–13 after resection, with a 5-year OS rate dropping to 58%. However, the subgroup of patients receiving ACT during weeks 11–13 differs somewhat from the other patients receiving ACT; they are generally older, more likely to live alone, and have undergone acute resections. These factors may contribute to delayed initiation of ACT and indicate a complex clinical profile that requires tailored management strategies.

### Strengths and limitations

A major strength of this study is the use of individual-level data from national registries known for their high quality and completeness. This allowed for the inclusion of crucial factors at the individual level in our analyses. As a result, our study provides unique insights and yields broadly representative results.

This study has some limitations. ACT was identified based on chemotherapy codes, regardless of the type, duration, or completion of the treatment. Consequently, some cases might include atypical practices or palliative treatments. However, given the sample size and the exclusion of stage IV patients, the number of patients who might have received palliative chemotherapy is likely low and does not affect the results. Another limitation is the low sample size of certain subgroups within our analysis, especially when stratifying on cancer risk level. The smaller subgroup sizes may have limited our ability to detect significant differences to these specific populations. The lack of significant survival benefit in high risk patients who received ACT in weeks 7–8 after resection can probably be explained by this factor.

## Conclusion

Our findings support initiation of ACT within 8 weeks after major resection to maximise survival benefits in elderly patients with stage III colon cancer. However, for certain patient groups, initiation of ACT seems beneficial even up to 10 weeks after resection. The findings suggest greater flexibility in ACT initiation timing, benefiting both patients and health care services.

## Data Availability

The data underlying this article cannot be shared publicly due to legal restrictions.

## Abbreviations

ACT: Adjuvant Chemotherapy
CI: Confidence Interval
CRN: Cancer Registry of Norway
FI: Frailty Index
HR: Hazard Ratio
ICD-10: International Classification of Diseases 10th Revision
ICPC: International Classification of Primary Care
OS: Overall Survival
SES: Socioeconomic Status
TNM: Tumour-Node-Metastasis
